# Accuracy of virtual surgical planning in mandibular reconstruction: application of a standard and reliable postoperative evaluation methodology

**DOI:** 10.1186/s12903-023-02811-8

**Published:** 2023-02-22

**Authors:** Yehia El-Mahallawy, Hams H. Abdelrahman, Haytham Al-Mahalawy

**Affiliations:** 1 Oral and Maxillofacial Surgery Department, Faculty of Dentistry, Alexandria University, Champlion St, Azrite, Alexandria Egypt; 2 Dental Public Health and Pediatric Dentistry Department, Faculty of Dentistry, Alexandria University, Alexandria, Egypt; 3 Oral and Maxillofacial Surgery Department, Faculty of Dentistry, Fayoum University, Fayoum, Egypt

**Keywords:** Mandibular reconstruction, Surgery, Computer assisted, Computer-aided design, Computer-aided manufacturing, Data accuracy, Software

## Abstract

**Objective:**

The purpose of this study was to determine the accuracy of virtual surgical planning for mandibular reconstruction, along with the implementation of a postoperative evaluation methodology.

**Materials and methods:**

The study is a prospective case series for computer-assisted mandibular reconstruction surgery. Analysis of the degree of agreement between virtual measurements and postoperative actual outcomes was performed. The reliability of the proposed evaluation methodology was assessed and analyzed using the Inter-Class Coefficient (ICC) test. Statistical significance was set at the 5% level.

**Results:**

Nine consecutive patients were selected. The analysis of all angular and linear parameters reported a highly statistically significant degree of agreement between the preoperative and postoperative measurements (*P* < 0.001). Furthermore, an extreme degree of reliability was reported when the evaluation methodology was scrutinized (ICC = 0.9).

**Conclusion:**

The excellent degree of agreement between the virtual plan and the actual outcome reported in this study validated the surgical accuracy of virtually assisted mandibular reconstruction. This study pointed out the reliability and reproducibility of the standardized evaluation protocol in an attempt to obtain a tolerable value for the acceptable postoperative results regarding the accuracy of computer-assisted surgery.

## Introduction

Segmental mandibular defects breed a drastic aesthetic and functional morbidity with highly demanding reconstructive goals that require the utmost degree of surgical fidelity. The preeminent objective of mandibular reconstructive surgery is to create a functional orthognathic result with a centric condyle position, along with aesthetic and symmetrical restoration of the form of the lower third of the face. The innate peculiar horseshoe mandibular bone configuration makes mandible reconstruction an arduous and research-demanding topic with considerable challenges for surgeons [1, 2].

Since its introduction in 2009 by Hirsch, Computer-Assisted Surgery (CAS) has become an integral part of mandibular reconstructive surgeries [3]. CAS offered a plethora of leverage over the conventional free-hand technique, offering more effective and predictable reconstruction outcomes. Virtual Surgical Planning (VSP) provides the surgeon with a digitalized platform on which he can predict, anticipate, and prevent surgical complications [4–7]. The contemporary implantation of digital technologies has vastly improved the quality of oral reconstruction and dental rehabilitation [7–10].

Rodby et al. depicted CAS in reconstructive surgery as a four chronologically phased operation. The first phase consists of a virtual surgical planning, followed by a Three-Dimensional (3D) modeling step. The third phase involves the transfer of the virtual design intraoperatively, or the surgical phase. The final stage of CAS involves postoperative evaluation, which despite being an integral part, it is usually overlooked [11–13]. In a systematic review on the topic of CAS accuracy in mandibular reconstruction, van Baar et al. concluded that there is a lack of homogeneity in the evaluation methodology that prohibited a meta-analysis calculation [14].

Despite being the workhorse option for mandibular reconstruction, vascularized fibular osteo-myo-cutaneous free flaps have considerable shortcomings that would not guarantee optimal results in every situation [15]. Vascularized free tissue transfer and microsurgical procedures put heavy emphasis on the resources of the medical sector, long operation period, and a greater financial load on the patient. Furthermore; a trained microvascular operator scarcity is usually a problem. Donor site morbidity and bone adequacy are further drawbacks for this kind of reconstruction procedure [15].

Anterior Iliac Crest Graft (AICG) provides a leading option in mandibular reconstruction, notably for lateral mandibular defects [15–17]. AICG bestows an easily accessible, dependable, and versatile harvesting site that yields an adequate osseous bulk and contour for three-dimensional defect reconstruction [18, 19]. Moreover, it has the unique ability to reconstruct both basal and alveolar bone providing vertical augmentation and bone quality of choice if osseointegrated implants are to be considered. Recipient bed vascularity, adequacy of investing soft tissue, defect size, donor site availability, and microvascular facility and experience availability are the main factors that favor the utilization of the AICG as a reconstructive alternative. An 83% success rate is reported by Bradley et al. with AICG implemented in mandibular defects less than 7 cm [20]. Although the indexed literature contains a plethora of virtual planning accuracy critiques, the postoperative evaluation of CAS accuracy in mandibular reconstruction with iliac graft is poorly reported [5, 12, 14, 18].

### Aim

This study was implemented to delineate the accuracy of the virtually planned mandibular reconstructive surgery with an iliac graft. Analysis of the degree of agreement between the actual postoperative measurements and the preoperatively virtually planned with CAS was the main intention of the study. The specific aims were to introduce the utilization of a standard accuracy assessment approach in the postoperative evaluation phase of CAS, with the deployment of a new statistical analysis methodology. Furthermore; the study examines the reliability and reproducibility of the utilized VSP evaluation methodology.

## Materials and method

### Study design

The 3D accuracy of virtually guided mandibular segmental defect reconstruction using AICG in comparison to the preoperative VSP was evaluated in a prospective case series. The sample size was calculated to be 9 patients estimated assuming 5% alpha error and 80% study power using a one-sample t-test to compare the mean to a null value = zero (Gpower 3.0.10).

Participants were recruited from the cases admitted to the Outpatient Clinic of Alexandria University Teaching Hospital, suffering from a disorder that necessitates segmental mandibular continuity defect reconstruction using AICG. The selected mandibular defects were planned for either immediate (primary) or delayed (secondary) reconstruction. Only patients of age were enrolled and excluding those with mandibular defect involving the condyle, or with a composite mandibular defect. Furthermore, Medically compromised patients, those requiring postoperative adjunctive therapy, those with a previous history of operations or injuries in the groin and iliac region, and those with active infection were excluded from the study. The study was conducted based on the Helsinki Declaration guidelines. Ethical Committee approval from the local Research Ethics Committee (IRB NO: 00010556-IORG: 0,008,839) was granted before the commencement of the study. All patients signed an informed consent before enrollment in this study.

### Preoperative virtual surgical planning

- Data acquisition

The CAS protocol was applied for all of the enrolled cases. All patients were treated at the Maxillofacial Unit of Alexandria University Hospital, Egypt. VSP commences with radiographic examination with Multi-Detector Computed Tomography (MDCT) scan (Philips Brilliance 64 MDCT, Philips, Eindhoven, Netherlands) for the maxillofacial (slice thickness 0.6 mm) as well as the pelvic region (slice thickness 1.0 mm). The Digital Imaging and Communications in Medicine (DICOM) data from the preoperative scans were obtained and fed to a specialized software where thresholding and segmentation of the bony tissue were performed to create a high-quality three-dimensional visualization in the form of a bone model (Mimics; Materialise, Leuven, Belgium). Proper visualization of the lesion edges and determination of the safety margin was set to create virtual proximal and distal osteotomy lines. Correspondingly, a bone model for the iliac region was also obtained.

- Rapid prototyping and templets designing

The mandible and the iliac bone models are imported to a 3D-planning software (3Matic; Materialise, Leuven, Belgium) in a Standard Tessellation Language (STL) format. Using the MDCT for verification of the osteotomy planes, a *Mandible Resection-Osteotomy Guide* was designed with a parameter offset of 2 mm, along with a *Reconstruction-Fixation Template* which maintains the 3D spatial relation between the proximal and distal segments after lesion resection and transfers this relation into the operation room. The screw boreholes in both the resection and fixation templates share exact position correspondence.

A mirroring tool based on the patient's mid-sagittal plane was utilized to create a *Neo-Mandible Model* with a symmetrical shape without the occurrence of lesion irregularities on the affected side. This was followed by the superimposition of the 3D reconstructed iliac bone on the site of the resected lesion in the *Neo-mandible*. Areas in the 3D iliac bone model with the best fit to the mandibular defect silhouette and matching the curve of the mandible are outlined to generate a *Harvesting Iliac Osteotomy Guide*. Union of the selected iliac contour along with the mirrored mandible was carried out to design the *Virtually Reconstructed Preoperative Mandible (VPM).*

The up-stated templates and VPM are exported in an STL format to a specialized 3D-printing software (NETFAB, Autodesk, CA, USA), and Fused Deposition Modelling (FDM) printing of the parts was performed. Pre-adaptation of the reconstruction plate was performed on the VPM, ensuring at least three screw holes in each bone stump. Templets were sterilized, following the Center for Disease Control (CDC) guidelines.

### Surgical procedures

An accompanying two-team approach was used in all of the enrolled patients. The first team of surgeons prepared the recipient area in the mandible, while the second elevated the iliac crest bone graft. The teams always consisted of the same surgeons. The mandible is exposed via the second neck crease incision, followed by fixation of the *Mandible Resection-Osteotomy Guide* via 2.0 mini-screws on the inferior border of the mandible. The proximal and distal osteotomies are marked followed by resection of the affected part of the mandible. The resection guide was replaced with the *Reconstruction-Fixation Template*, which was fixed to the remaining mandibular stumps at the boreholes created for the fixation of the resection template. The fixation template will maintain the spatial relation between the bony stumps which enabled the placement of the *Pre-Adapted Reconstruction Plate*. Concurrently, the second team exposed the tri-cortical configuration of the iliac crest and the *Harvesting Iliac Osteotomy Template* was fitted to the anatomy of the iliac tubercle, where the harvesting margins were drawn according to the template’s outline. This was followed by graft placement in the proper position and rigidly fixed with the reconstruction plate.

### VSP accuracy evaluation and statistical analysis

**Fig. 1:**
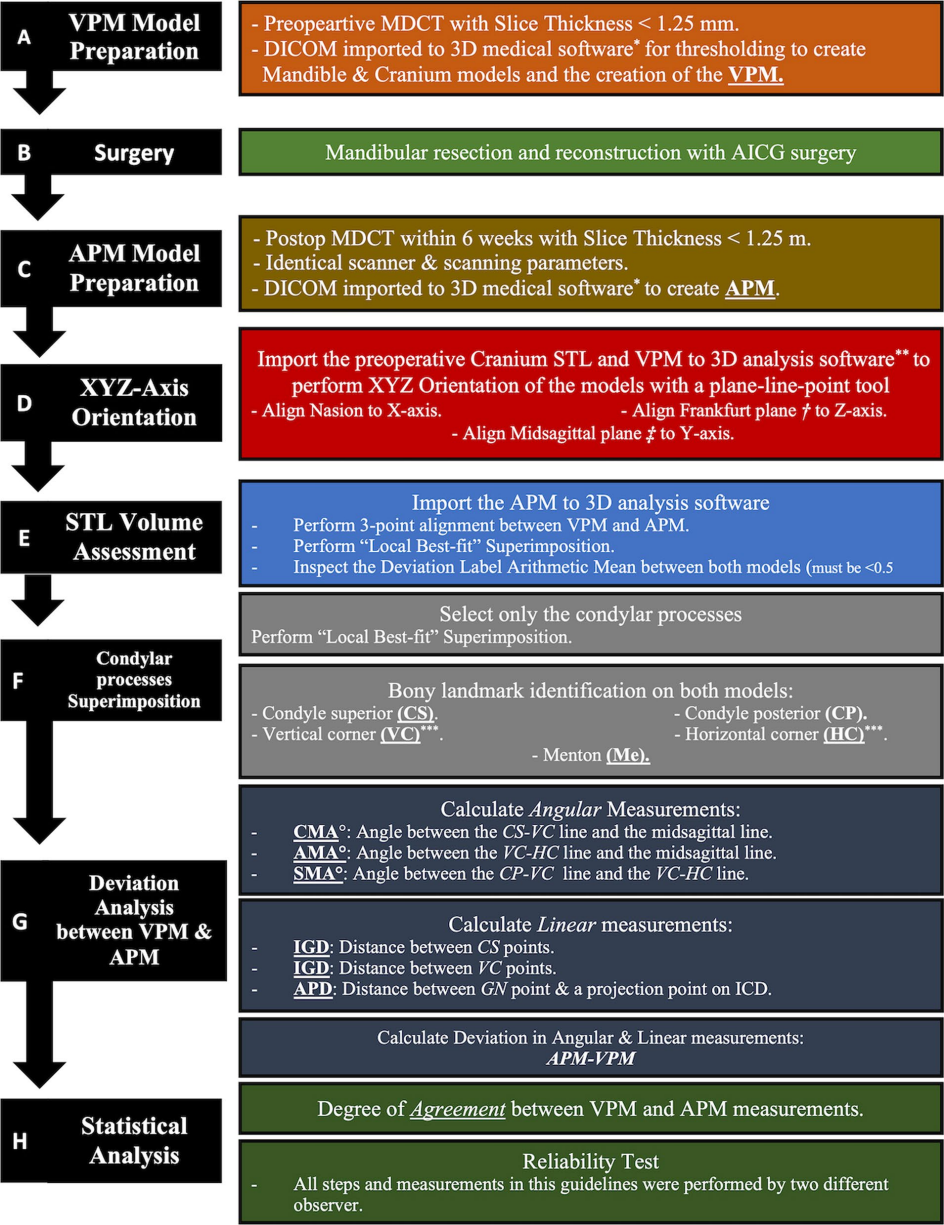
A Descriptive colour-coded flowchart of the CAS mandibular reconstruction steps and the proposed postoperative accuracy evaluation methodology. * Mimics software, **GOM Inspect Pro, ***Angle corner according to Brown et al. classification ^2^, ****Canine or 7 mm anterior to mental foramen according to Brown et al. classification ^2^, ^†^ Uppermost point of the external auditory meatus and most inferior point of the left orbital rim, *‡* Nasion, Basion, Incisive Foramen

**Fig. 2:**
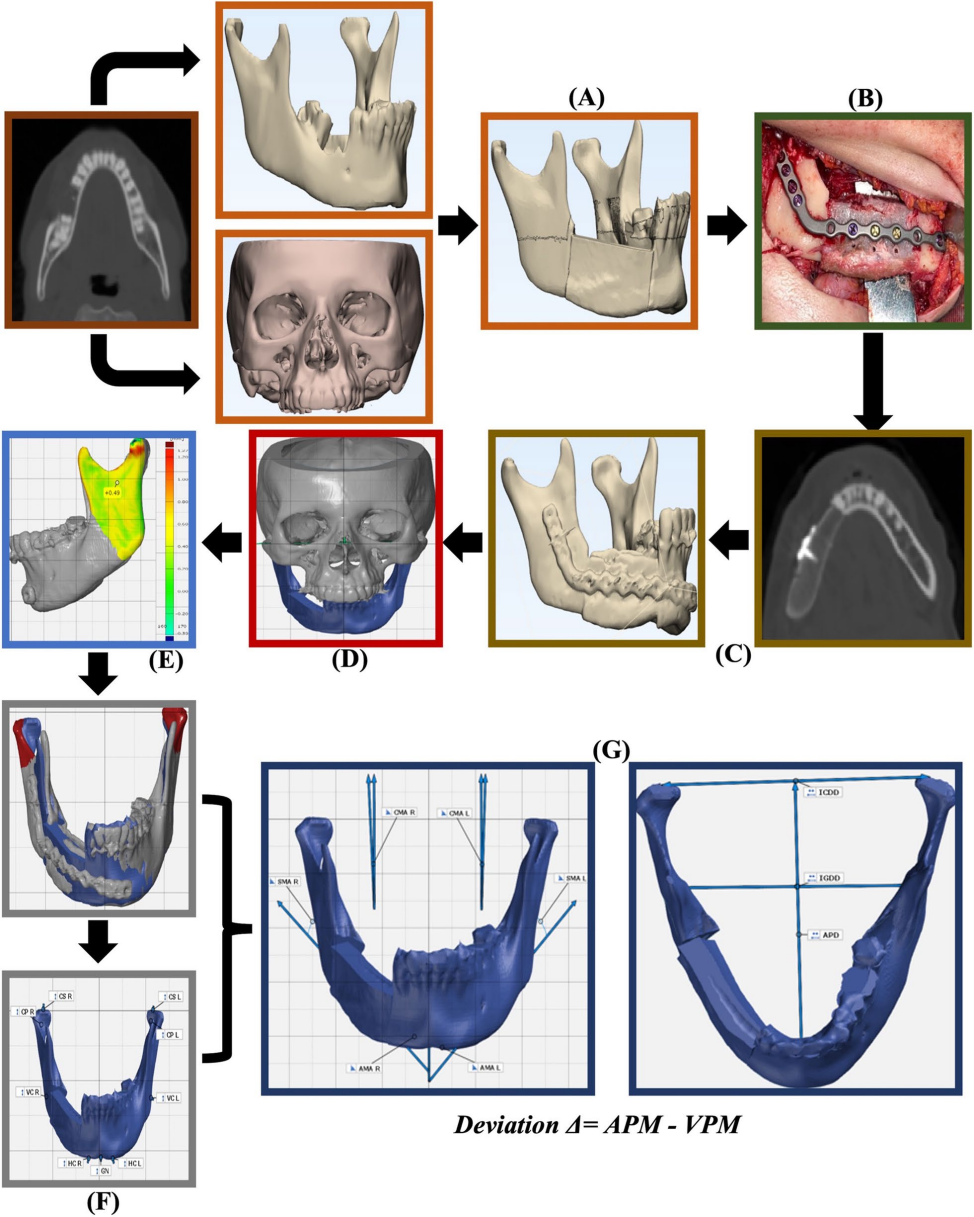
An illustrative colour-coded, descriptive images flowchart of the CAS mandibular reconstruction steps and the proposed postoperative accuracy evaluation methodology. **A** VPM model preparation. **B** Surgery. **C** APM model preparation. **D** XYZ-Axis orientation. **E** STL volume assessment. **F** Condylar process superimposition. **G** Deviation analysis between APM and VPM

**Fig. 3:**
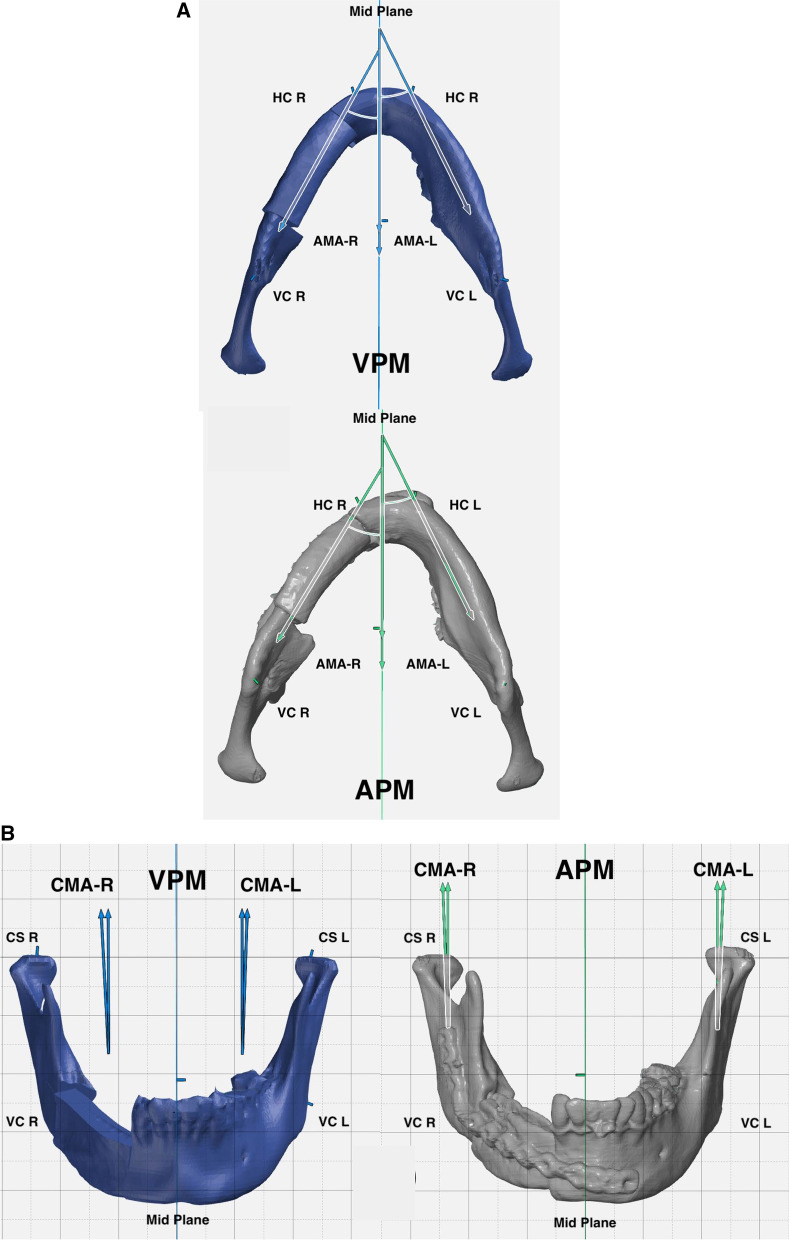

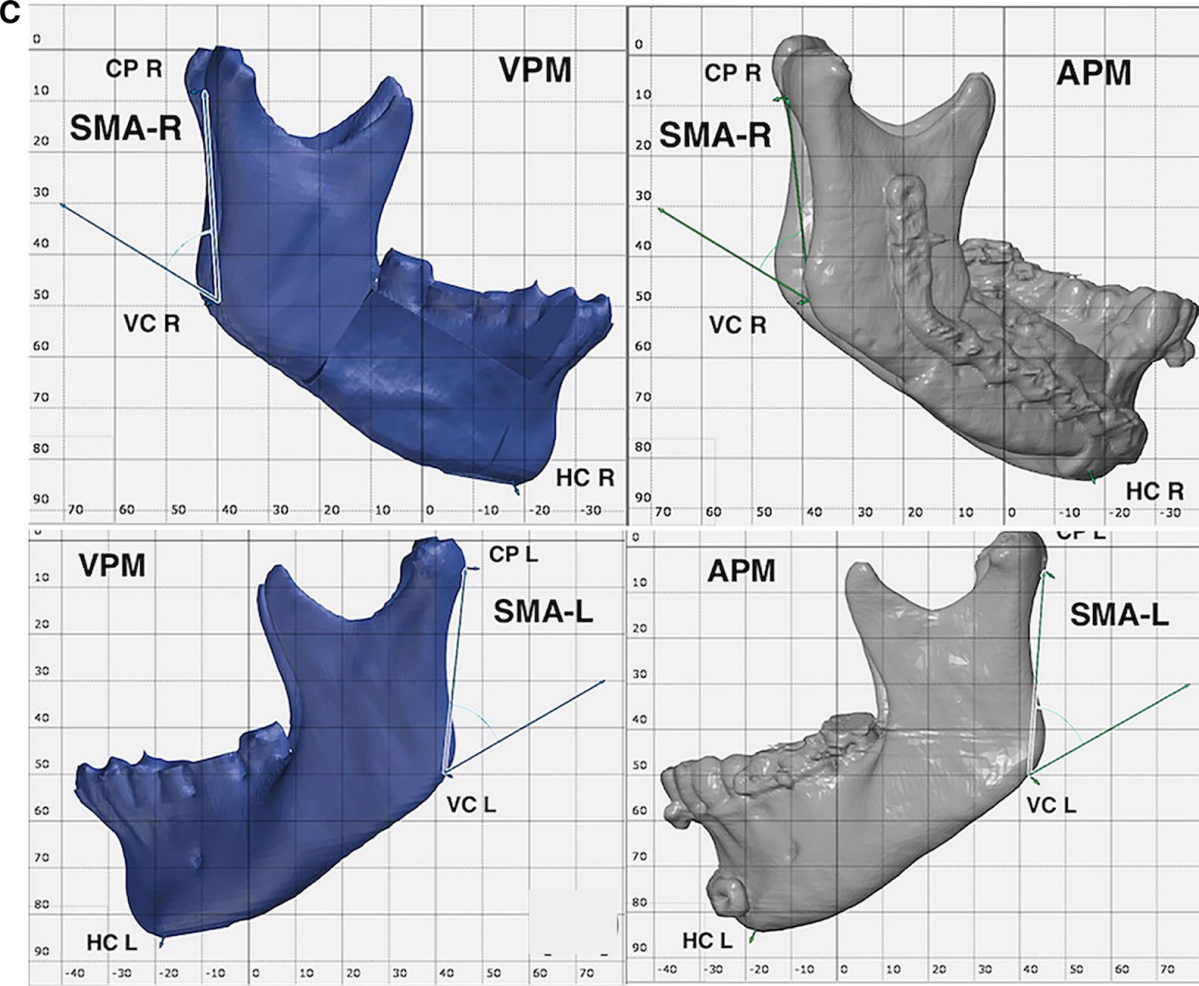
Demonstration of Angular mandibular analysis. **A** preoperative and postoperative Axial mandibular angles. **B** preoperative and postoperative Coronal mandibular angles. **C** preoperative and postoperative Sagittal mandibular angles

**Fig. 4:**
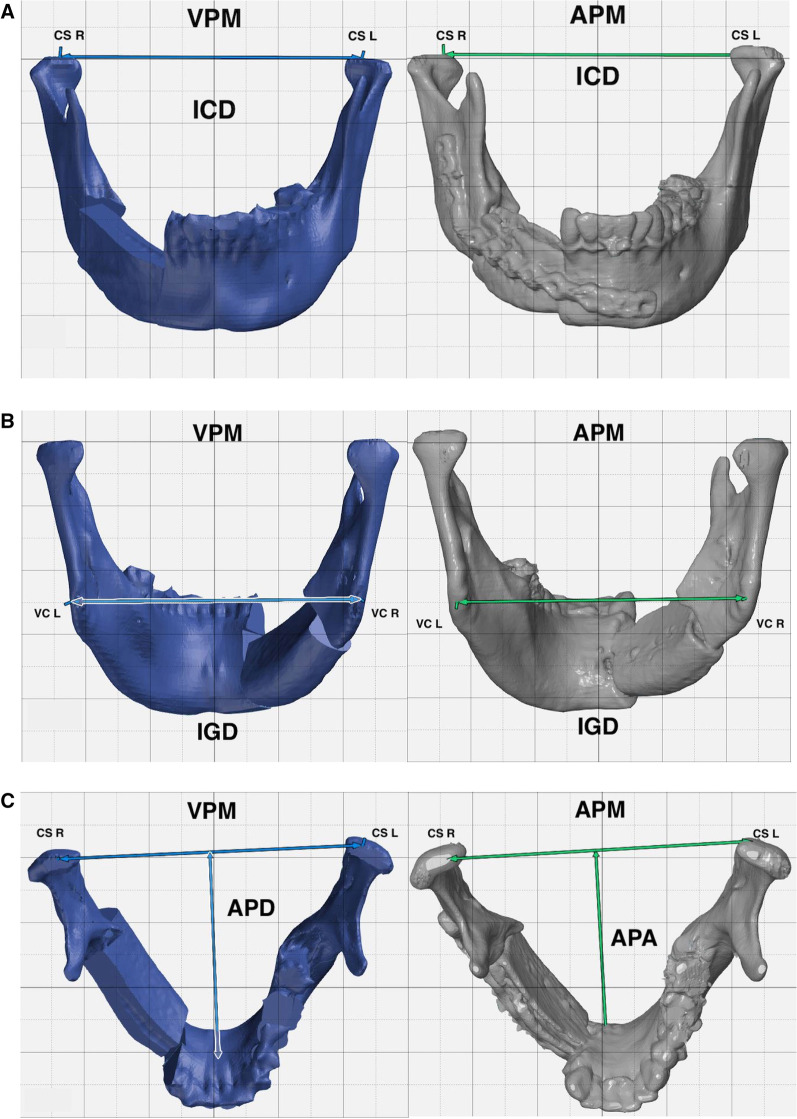
Demonstration of Linear mandibular analysis. **A** preoperative and postoperative Inter-Condylar distance. **B** preoperative and postoperative Inter-Gonial distance. **C** preoperative and postoperative Antero-Posterior distance

For each patient, a postoperative MDCT scan of the maxillofacial region was obtained within 7 days of the surgery using the same preoperative scanning parameters. An *Actual Postoperative Mandible* (APM) model was segmented from the DICOM file in a similar manner, which will be then exported along with the preoperative *VPM* model to a 3D-analysis software (GOM Inspect Pro 2019; GmbH, Braunschweig, Germany). The APM was correlated to the VPM according to the evaluation guidelines proposed by Van Baar et al. [21, 22]. A description of the proposed guidelines flowchart is presented in Fig. 1 with an illustrative flowchart in Fig. 2. An amendment to the solely angular measurements in the proposed guidelines was used to correlate the accuracy analysis to the functional outcome. Deviation analysis for three linear dimensions was incorporated to determine if the planned orthognathic result was achieved; *Inter-Condylar Distance* (ICD), *Inter-Gonial Distance* (IGD), and *Antero-Posterior Distance* (APD) (Figs. 3,4).

Data were analyzed using IBM SPSS for windows version 23.0. (IBM Corp, NY, USA). Deviation in measurements was presented using absolute mean, range, and standard deviation. Agreement between the preoperative VPM measurements and the postoperative APM measurements was scrutinized using a two-tailed Intra Class Correlation Coefficient (ICC) test. A key to apprehending the outcome values of the ICC is presented as follows: < 0.5 Poor agreement, 0.5 to < 0.75 Moderate agreement, 0.75 to < 0.9 Good agreement, 0.9—1.0 Excellent agreement [23]. The significance level was confirmed at a *P* value of 0.05.

### Reliability of the measurement’s guidelines

To assess the ease of application of the proposed guidelines and the reliability of computer-based measurement, each patient dataset was evaluated by two separate investigators (Y.E, H.A). A demo case was used for training and orientation of the investigators on the applications of the guidelines, where their data was not included in this study. Inter-observer reliability was inspected by a two-way mixed ICC test to determine the degree of conformity between the iterations of two separate auditors. The significance level was confirmed at a 5% level.

## Results

Nine patients were enrolled in this study. An abridged tabulation of the patient’s demographic data is presented in Table 1. Demographic input revealed a male-to-female ratio of 0.5:1, with a mean age of 37.4 ± 12.01 years.

**Table 1:**
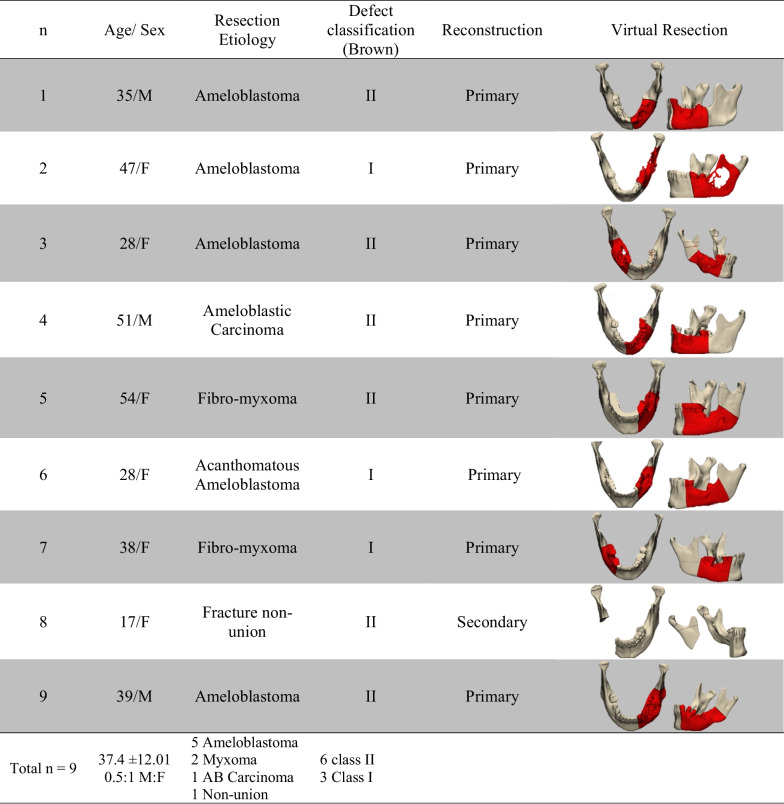
Abridged patients demographic data

n, Number; yr, Year; M, Male; F, Female; M:F, Male: Female Ratio

Table 2 describes the appraisal of the angular deviation between the preoperative VPM and the postoperative APM measurements. The maximum degree of deviation was 4.24**°** in the Sagittal Mandibular Angle (SMA), while the smallest difference was reported in the right Coronal Mandibular Angle (CMA) (0.02**°**). The absolute mean liner deviation regarding the ICD, IGD, and APD distance is tabulated in Table 3.

**Table 2:** Absolute mean angular deviation (°) (APM-VPM)

Δ/°	AMA		CMA		SMA	
	R	L	R	L	R	L
Mean ± SD	1.50 ± 1.07	0.95 ± 0.51	0.57 ± 0.68	0.96 ± 0.76	2.35 ± 1.17	2.13 ± 1.07
Min—Max	0.38 – 0.64	0.10 – 1.86	0.02 – 2.22	0.14 – 2.27	0.64 – 4.23	0.32 – 4.24

Δ, Deviation; APM, Actual Postoperative Model; VPM, Virtual Preoperative Model; AMA, Axial Mandibular Angle; CMA, Coronal Mandibular Angle; SMA, Sagittal Mandibular Angle; R, Right Side; L, Left Side; SD, Standard Deviation; Min, Minimum value; Max, Maximum Value

**Table 3:** Absolute mean linear deviation (mm) (APM-VPM)

Δ/mm	ICD	IGD	APD
Mean ± SD	0.24 ± 0.18	1.11 ± 0.93	1.02 ± 0.60
Min—Max	0.04 – 0.49	0.35 – 3.41	0.34 – 2.06

Δ, Deviation; Actual Postoperative Model; VPM, Virtual Preoperative Model; ICD, Inter-Condylar Distance; IGD, Inter-Gonial Distance; APD, Antro-Posterior Distance; SD, Standard Deviation; Min, Minimum value; Max, Maximum Value

The agreement between APM and VPM measurement was ratified using the ICC. An excellent agreement was found in all of the linear measurements (ICC value 0.9 to 1). Concurrently a good agreement (0.75 to < 0.9) was computed in the right Axial Mandibular Angle (AMA) and both CMA. All of the remaining angular measurements once again revealed an excellent agreement. For both the linear and the angular measurements, a statistically significant correspondence was found between the obtained actual postoperative measurements and the preoperatively virtually planned ones. (Least reported *P* = 0.012^*^, and the highest is *P* < 0.0001^*^) (Tables 4, 5).

**Table 4:** Analysis of the degree of agreement between the APM and the VPM Angular measurements

	AMA		CMA		SMA	
	R	L	R	L	R	L
ICC	0.863	0.978	0.841	0.894	0.984	0.987
*P*	< 0.0001*	< 0.0001*	0.012*	< 0.0001*	< 0.0001*	< 0.0001*

Actual Postoperative Model; VPM, Virtual Preoperative Model; AMA, Axial Mandibular Angle; CMA, Coronal Mandibular Angle; SMA, Sagittal Mandibular Angle; R, Right Side; L, Left Side; ICC, Interclass Correlation Coefficient

ICC Outcome Values: < 0.5 Poor agreement, 0.5 to < 0.75 Moderate agreement, 0.75 to < 0.9 Good agreement, 0.9—1.0 Excellent agreement

^*^Statistically significant difference at *p* value ≤ 0.05

**Table 5:** Analysis of the degree of agreement between the APM and the VPM Linear measurements

	ICD	IGD	APD
ICC	0.987	0.979	0.988
*P*	< 0.0001*	< 0.0001*	< 0.0001*

Actual Postoperative Model; VPM, Virtual Preoperative Model; ICD, Inter-condylar Distance; IGD, Inter-Gonial Distance; APD, Antro-Posterior Distance; ICC, Interclass Correlation Coefficient

ICC Outcome Values: < 0.5 Poor agreement, 0.5 to < 0.75 Moderate agreement, 0.75 to < 0.9 Good agreement, 0.9—1.0 Excellent agreement

^*^Statistically significant difference at *p* value ≤ 0.05

An appraisal of the guideline’s reliability was performed for both the angular and liner measurements. An excellent agreement (ICC = 0.9–1.0) was observed between the measurements of both observers, bar from the CMA and ICD measurements which computed a good agreement (ICC = 0.75 to < 0.9). Accordingly, statistically significant inter-observer reliability was attained when performing these guidelines in the assessment of postoperative accuracy of the planned mandibular resection and reconstruction with AICG (Table 6).

**Table 6:** Intra Examiner Reliability of the measurements made by the main observer and the other observer

	AMA		CMA		SMA		ICD	IGD	APD
	R	L	R	L	R	L			
ICC	0.972	0.947	0.841	0.888	0.949	0.976	0.785	0.954	0.948
*P*	< 0.0001*	< 0.0001*	0.012*	0.004*	< 0.0001*	< 0.0001*	0.027*	< 0.0001*	< 0.0001*

AMA, Axial Mandibular Angle; CMA, Coronal Mandibular Angle; SMA, Sagittal Mandibular Angle; SD, Standard Deviation; R, Right Side; L, Left Side; ICC, Interclass Correlation Coefficient

ICC Outcome Values: < 0.5 Poor agreement, 0.5 to < 0.75 Moderate agreement, 0.75 to < 0.9 Good agreement, 0.9—1.0 Excellent agreement

^*^Statistically significant difference at *p* value ≤ 0.05

## Discussion

A plethora of reports are available regarding the accuracy of VSP with fibular flaps, yet the literature comes short in the number of manuscripts that evaluates the accuracy of mandibular reconstruction with AICG [5, 12, 14]. Evaluation of postoperative accuracy is one of the integral phases of CAS which is usually forgotten [11]. Hence, this study evaluated the accuracy of CAS for mandibular reconstruction with AICG. Furthermore, this study intended to implement a standard guideline for the postoperative CAS accuracy evaluation and determine its degree of reliability.

The bizarre and abrupt angulations in the mandibular bone make its reconstruction more difficult and with a significantly perceptible effect on facial harmony when reconstruction inaccuracies occur [24]. Hence, the assessment of angular deviation is of significant implications for the morphological outcome of the reconstruction [24]. De Maesschalck et al. first introduced the assessment of CAS hard tissue accuracy by the evaluation of angular deviations between the Virtual model (VPM) and the Actual Model (APM), acquiring reproducible results while bypassing the superimposition errors [25]. In their report, De Maesschalck introduced the concept of axial, coronal, and sagittal mandibular angles. Their report calculated mean angular deviation values of 1.0°, 1.8°, and 4.2° for each mandibular angle. They deemed their outcome by CAS as morphometric accurate [25]. This was modified by van Baar et al. by the adoption of the vertical and horizontal corners defined by Brown et al. in their comprehensive classification [2, 21]. This modification was in an attempt to standardize the evaluation method and make it reproducible [21]. The angular deviation was also utilized by Weitz et al., where a range of 0°–18° degree of deviation was calculated [26]. In a systematic review regarding the accuracy of CAS in mandibular reconstruction, angular deviations were considered in 17 cases, and the range of reported postoperative deviation results was 0.9° and 17.5° [14]. Furthermore, and to our knowledge, the implantation of angular deviation assessment to determine the accuracy of computer-aided AICG mandibular reconstruction has not been described previously.

In the only published report where a standardized evaluation methodology was utilized, van Baar et al. disclosed ranges of mandibular angles in the evaluation of the accuracy of CAS [27]. They reported deviation in the AMA that ranged from 0.12° to 6.42°, and a range of 0.06° to 2.99° was calculated in the CMA and SMA deviation range of 0.1° to 7.47°. In their report, they deemed those values as accurate with no extreme deviations [27]. In this study, a nearly identical agreement was found in the CMA range (0.02° to 2.27°), while much lower AMA and SMA ranges were calculated (0.10° to 1.86°, and 0.32° to 4.24° respectively). Although this report owes a higher sample, the difference may be contributed to the complexity of the cases. In the up-mentioned report, there was a larger number of Brown class III cases, whereas no class III were reported in this study as the nature of the graft limits its application in more complex defects. The application of angular deviation is an easy method to determine the overall morphology of the lower third of the face with few numerical inputs, unlike the linear deviation. A similar conclusion could be used with fewer angular parameters than with linear ones.

Linear deviation analysis is a trivial method to evaluate the accuracy of the VSP used in mandibular reconstruction, however, the majority of the reports are concerned with fibular bone flaps [4, 13, 24, 28]. It is usually represented as two transverse dimensions, ICD and IGD, and one sagittal dimension, APD. Foley et al. studied the linear deviation of CAS mandibular reconstruction utilizing AICG. Their study calculated a 1.7 mm deviation in the APD, 1.6 mm in IGD, and 0.2 mm in ICD [18]. In this study, ICD deviation computed an absolute mean value of 0.24 ± 0.18 mm, in comparison to 1.11 ± 0.93 mm regarding the IGD, and APD difference of 1.02 ± 0.60. These values are comparable to those reported in the literature [18, 29–31]. Analysis of linear deviations provides a simple means to correlate surgical accuracy to functional outcome in ICD, and morphological outcome, in IGD and APD.

Postoperative accuracy analysis studies are limited by the fact that they evaluate only bony reconstruction in contrast to a more complex procedure that is affected by several other components, such as surgery effects, soft tissue reconstruction, adjunctive therapy application, and dental rehabilitation [14, 32]. This makes it difficult to statistically correlate surgical accuracy with optimal functional outcomes owing to the vast confounding factors [14, 32]. Nearly all of the CAS evaluation studies lack a statistical analysis of their outcome, they only just deemed the reconstruction as accurate [25, 26, 33]. In this study, we performed an ICC test to determine the degree of agreement between the VSP measurements and the actual postoperative ones, where all of the linear and angular measurements revealed a statistically significant degree of agreement. This statistical analysis may be a more comprehensible way to define the degree of accordance between the actual outcome and the virtual plan and overlook minor deviations as negatable values. The statement of more identical outcomes makes the measured results more valid for further analyses than testing the degree of error between the data.

The utilization of the postoperative evaluation methodology in this study revealed a statistically significant inter-observer reliability (ICC = 0.9–1.0), which reveals the reproducibility of the utilized guidelines in the assessment of computer-assisted mandibular reconstruction with AICG. The lack of uniformity in the accuracy studies leads to few tests of reliability performance in the literature [34–36]. Ritschl et al. reported very good intra- and interobserver reliabilities for horizontal linear deviation in an in-house, open-source computer-planned mandibular reconstructions with fibular flap [36]. Despite yielding similar outcomes [34–36], this cannot be correlated to the results of this study owing to the diverse methodology. However, this may validate the use of the STL model comparison and deviation as a reliable and reproducible accuracy assessment technique.

CAS in orthognathic surgeries has a more uniform postoperative evaluation method which yielded a reasonable cut-off value for linear deviation at 2 mm, where it is stated that these deviation values are not likely to be perceived by the naked eye [37, 38]. In the reconstruction field, several authors demonstrated the lack of comparable data in the literature owing to the vast heterogenicity in the CAS evaluation methodology [14, 32]. The application of standardized guidelines may be the first step in determining the tolerable range for mandibular reconstruction outcomes. This study's deviation outcome values are in context with most of the reported results in the literature, despite the significant heterogeneity in their methodologies.

The neglectable and insignificant angular and linear deviation in the postoperative scan when compared to the virtual scheme delineates the accuracy of the performed surgery in recreating the virtual preoperative situation. Furthermore, a conjunction of several of these measurements could be interrupted as an exemplary morphological and functional performance of the operation.

Despite the notion of optimal accuracy, Inaccuracies in the postoperative measurements are normal and well apprehended. Several causative elements may cause this minor deviation along various steps of VSP [14, 32]. van Eijnatten et al. report that all tomography-based STL models displayed non-uniform volumetric deviations of up to 0.9 mm [39]. Cone Beam Computed Tomography (CBCT) scanners exhibit the greatest STL model volume deviation, while MDCT provides the least degree of reported geometric deviation [22, 39]. Huang et al. documented the vast deviation that can occur with changes in CT acquisition and reconstruction parameters, especially slice thickness and pixel size. A bigger slice thickness brings in an inaccurate STL model with lost details which influences the accuracy and fitting of 3D-printed templates [40]. Whyms et al. conducted that accurate volumetric measurements were obtained at the cut-off slice thickness of 1.25 mm [41]. The main error in the evaluation phase occurs during bony landmarks localization [14, 42]. In a systematic review regarding the postoperative accuracy evaluation of CAS, few reports in the literature implemented the orientation of the STL models to the XYZ axis [14]. Alignment of the preoperative STL models to the XYZ axis acts as a benchmark from which any angular or linear deviations can be computed with great uniformity and reproducibility. The employment of an easy to perform and clear guidelines is important to add validity to the outcome of the accuracy studies [18, 34]. Standardization of the evaluation methodology will not yield an identical outcome, yet it aims at providing uniformity to the technique. In this study, the proposed evaluation methodology was implemented, where all of the enrolled patients had a standardized CT image acquisition parameter in the preoperative and postoperative scans.

Virtual planning gives the surgeon the added leisure to anticipate complications preoperatively which, along with the pre-preparation of the fixation plate, drastically decreases the operation time and improves intraoperative execution. The use of CAS also leads to precision regarding the accuracy of stump-graft contact, maximizing the success of the grafting procedure. [14, 32, 43]. A handful of advantages for VSP in mandibular reconstruction are documented in the indexed literature and their discussion is beyond the scope of this article. Despite that, the literature lacks uniformity about a clear standard for CAS as it is surgeon/engineer experience-based [14, 32, 43]. The added leverages of the CAS almost always overthrow its drawbacks. This study further added confirmation regarding the accuracy of CAS in mandibular reconstruction with AICG, which falls in line with the literature consensus about surgical outcomes.

## Conclusion

The excellent degree of agreement between the virtual and actual measurements indicates the exemplary bony accuracy of the CAS in the surgical mandibular reconstruction with an iliac graft. The application of standardized evaluation guidelines with this novel statistical analysis could be a milestone in the attempt to standardize the evaluation criteria and obtain a tolerable value for acceptable postoperative results of mandibular reconstruction outcomes. Furthermore, the study pointed out the ease of application and reproducibility of the utilized accuracy evaluation protocol. Future studies with the same methodology are in need to disclose a meta-analysis about computer-assisted mandibular rehabilitation with various reconstructive options.

## Data Availability

All data generated or analysed during this study are included in this published article.

## Abbreviations

CAS: Computer-Assisted Surgery
VSP: Virtual Surgical Planning
3D: Three-Dimensional
AICG: Anterior Iliac Crest Graft
MDCT: Multi-Detector Computed Tomography
DICOM: Digital Imaging and Communications in Medicine
STL: Standard Tessellation Language
VPM: Virtually Reconstructed Preoperative Mandible
FDM: Fused Deposition Modelling
CDC: Canter for Disease Control
APM: Actual Postoperative Mandible
AMA: Axial Mandibular Angle
SMA: Sagittal Mandibular Angle
CMA: Coronal Mandibular Angle
ICD: Inter-Condylar Distance
IGD: Inter-Gonial Distance
APD: Antero- Posterior Distance
ICC: Intra Class Correlation Coefficient
